# Biliary peritonitis due to “fallen” hydatid cyst after abdominal trauma

**DOI:** 10.4103/0974-2700.41791

**Published:** 2008

**Authors:** Melih Kara, Deniz Tihan, Tuba Fersahoglu, Faruk Cavda, Izzet Titiz

**Affiliations:** 1 Haydarpaşa Numune Training and Research Hospital, 1^st^ General Surgery Clinic, Istanbul, Turkey; 2 Department of General Surgery, Istanbul University, Istanbul Faculty of Medicine, Istanbul, Turkey

**Keywords:** Biliary peritonitis, “fallen” hydatid cyst, trauma

## Abstract

Hepatic hydatid cysts may cause serious complications. Intraperitoneal rupture of hepatic hydatid cyst is rarely seen and the prognosis can be fatal. By experience, we know that it might be difficult to diagnose an unruptured cyst expulsed into the peritoneal cavity. In this report, we present the case of a 54-year-old man with an intraperitoneal cystic mass of 10 cm of diameter which had extruded out from the liver due to a blunt abdominal trauma.

Intraperitoneal rupture of hydatid cyst of the liver is a rare situation and the prognosis is usually fatal. However, expulsion into the peritoneal cavity without perforation is a much more unusual complication of a hepatic hydatid cyst. We report the observation of a 54-year-old man who was diagnosed of biliary peritonitis due to an unruptured hepatic hydatid cyst after a blunt abdominal trauma.

## CASE HISTORY

A 54-year-old man presented in the emergency unit (EU) of our hospital with severe abdominal pain, 2 days after falling down on his abdomen at home. Patient history was remarkable for previously diagnosed multiple hepatic hydatid cysts, and he had been on albendazole regimen for 1 month. He had no known disease other than a recent blunt abdominal trauma as he had fallen down on his abdomen 2 days prior to showing up in the EU. As he stated, he had never had a similar type of pain before this house accident, and the pain had started to ‘really hurt’ several hours after ‘the fall.’ Intensity of the pain was gradually increasing for 2 days.

**Figure 1:**
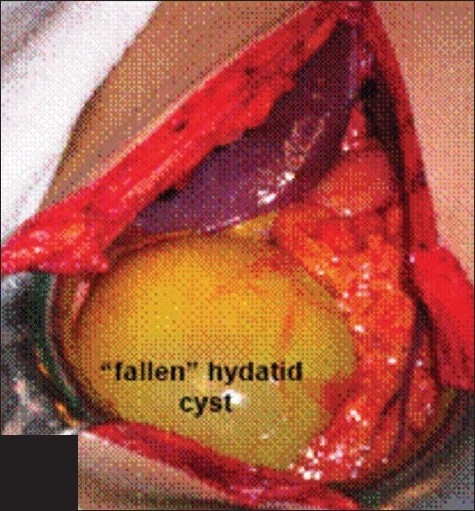
The cyst in the abdomen

Initial physical examination revealed diffuse abdominal tenderness on palpation with epigastric predominance and positive rebound tenderness at each abdominal quadrant. As per initial vital signs, arterial blood pressure: 140/90 mm Hg, heart rate: 96/min and the body temperature (axillary) was 37.5°C. Chest X-ray and acute abdominal X-ray series were normal. Abdominal ultrasonography showed three intrahepatic and one extrahepatic cystic masses [Figure 1], approximately 10 cm in diameter each. Intrahepatic cysts were seperately located in the second, fifth and seventh segments of the liver, where as the extrahepatic cyst was situated close to the left upper quadrant. There was also considerable amount of abdominal free fluid seen in the ultrasound. Laboratory tests revealed markedly increased white blood cell count (WBC: 23,000/mm^3^, eosinophils: 0.43 × 10³/µl). The patient's overall clinical and laboratory findings clearly suggested the diagnosis of acute abdomen and immediate surgical intervention was considered.

**Figure 2:**
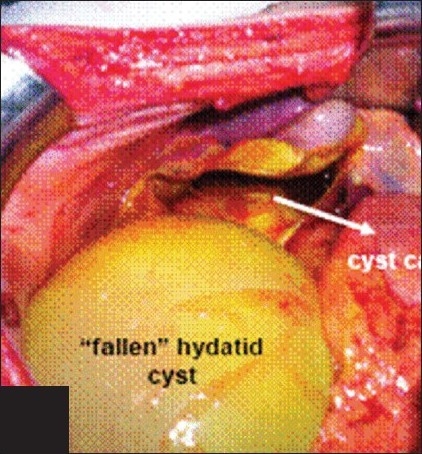
Bile leakage seen in the cystic cavity

Patient was promptly prepared and taken into the operating room. Abdominal exploration revealed considerable amount of serous free fluid with bile leakage in the abdomen. Sample fluid was spared for laboratory and remaining was aspirated. Further exploration was interesting as we found an unruptured hydatid cyst of 10 cm in diameter with an intact germinative membrane, which was enucleated spontaneously out from the fifth hepatic segment. We made this assumption since there was an entirely empty cystic cavity in the fifth segment with the same dimensions as the wandering extrahepatic cyst. Bile leakage from the wall of this cavity was clearly seen [Figure 2]. We used ligating sutures to control bile leakage. The unruptured cyst was taken out of the abdomen and the two other hepatic cysts were enucleated. We also performed cholecystectomy because the expulsed cyst's bed was in close proximity to the gallbladder. We did not further explore the choledochus. The patient was discharged 8 days after the operation without any complication. Chemotherapy with albendazole (10 mg/kg/day) was recommended for 6 months.

## DISCUSSION

Hydatid disease is not an uncommon problem, especially in endemic countries.[1] Human hydatid disease occurs by infestation with *Echinococcus granulosus* and less frequently with *Echinococcus multilocularis*.[1,2] In the normal life cycle of *Echinococcus* species, adult tapeworms inhabit the small intestine of carnivorous definitive hosts (dogs, wolves, and coyotes), and echinococcal cyst stages occur in herbivorous intermediate hosts (sheep, cattle and goats, pigs, horses, and camels). In the typical life cycle, tapeworm eggs are passed in the feces of an infected dog and ingested by grazing sheep; hatch into embryos in the intestine, penetrate the intestinal wall, and then carried by blood to major filtering organs (liver and/or lungs). Embryos localize in a specific site and they develop into larval echinococcal cysts where new tapeworm heads (protoscolices) are produced by asexual reproduction. Dogs are infected by ingesting viscera of home-slaughtered livestock containing echinococcal cysts. Protoscolices attach to the dogs' intestinal wall and develop into mature adult tapeworms. In primary echinococcosis, metacestodes develop from oncospheres after peroral infection. In secondary echinococcosis, larval tissue proliferates after being spread from the primary site (i.e. *via* rupture due to medical interventions or trauma). Most patients have single organ involvement with a solitary cyst, and two-thirds of the patients experience liver echinococcosis. Lung is the second most commonly involved organ. Cysts are surrounded by the parasitic host tissue called pericyst where larval endocysts are located. Outermost layer of the cyst is a hyaline membrane which covers the multipotential germinal layer where protoscolices and brood capsules are produced. Cystic cavity is filled with clear fluid, brood capsules, protoscolices and also daughter cysts are frequently found in the cyst. Symptoms of the disease are variable and depend on the cyst localization, size, complications caused by rupture, bacterial infection of cysts, spread into bile ducts or blood vessels, immunologic reaction such as membranous nephropathy, asthma, and anaphylaxis. Cystic echinococcosis is rarely fatal and death occurs because of anaphylactic shock or cardiac tamponade in case of cardiac echinococcosis. The incidence of rupture of the hydatid cyst of the liver is about 15-40% of the cases.[3] Trauma is a cause of hydatid cyst rupture and falls are the most common causes of blunt abdominal trauma.[4] Both ultrasonography and computerized tomography are very highly sensitive in demonstrating cyst rupture.[4] Perforation of the cyst into the peritoneal cavity can become dramatic since it progresses to acute abdomen and usually ends up with anaphylaxis.[5] However, traumatic intraperitoneal enucleation of a hydatid cyst without perforation as in our case, is a very rare condition. To our knowledge, there is only one similar case described in the literature. Sharma and Gupta reported a similar case with enucleation af a hydatid cyst of 20 × 20 cm^2^ diameter without rupture.[6] In our case, cystic dimensions were smaller. Intraperitoneal enucleation of hydatid cyst can also present as biliary peritonitis. Thus, in the endemic areas, this unusual condition should be kept in mind in the differential diagnosis of abdominal pain. Carefully obtained medical history of the patient, an attentive physical and radiological examination will be helpful for the correct diagnosis. Surgical intervention combined with albendazole therapy is still the best way in the treatment of these patients.

## References

[1] Sayek I, Yalin R, Sanac Y (1980). Surgical treatment of hydatid disease of the liver. Arch Surg.

[2] Langer JC, Rose DB, Keystone JS, Taylor BR, Langer B (1984). Diagnosis and management of hydatid disease of the liver: A 15-year North American experience. Ann Surg.

[3] El Malki HO, El Mejdoubi Y, Mohsine R, Ifrine L, Belkouchi A (2006). Intraperitoneal perforation of hepatic hydatid cyst. Gastroenterol Clin Biol.

[4] Gunay K, Taviloglu K, Berber E, Ertekin C (1999). Traumatic rupture of hydatid cysts: A 12-year experience from an endemic region. J Trauma.

[5] Di Cataldo A, Lanteri R, Caniglia S, Santangelo M, Occhipinti R, Li Destri G (2005). A rare complication of the hepatic hydatid cyst: Intraperitoneal perforation without anaphylaxis. Int Surg.

[6] Sharma BG, Gupta KK (2000). Spontaneous intraperitoneal expulsion of an unruptured hydatid cyst. Saudi Med J.

